# A Crack Propagation Method for Pipelines with Interacting Corrosion and Crack Defects

**DOI:** 10.3390/s22030986

**Published:** 2022-01-27

**Authors:** Mingjiang Xie, Yifei Wang, Weinan Xiong, Jianli Zhao, Xianjun Pei

**Affiliations:** 1 School of Mechanical Engineering, Southeast University, Nanjing 211189, China; mingjiang@seu.edu.cn (M.X.); yifeiwang@seu.edu.cn (Y.W.); 220210314@seu.edu.cn (J.Z.); 2 Department of Information System, Dalian Naval Academy, Dalian 116018, China; xiongweinan6616@163.com

**Keywords:** pipeline, fatigue crack, corrosion, stress intensity factor, finite element, XGBoost

## Abstract

Corrosion and crack defects often exist at the same time in pipelines. The interaction impact between these defects could potentially affect the growth of the fatigue crack. In this paper, a crack propagation method is proposed for pipelines with interacting corrosion and crack defects. The finite element models are built to obtain the Stress Intensity Factors (SIFs) for fatigue crack. SIF interaction impact ratio is introduced to describe the interaction effect of corrosion on fatigue crack. Two approaches based on extreme gradient boosting (XGBoost) are proposed in this paper to predict the SIF interaction impact ratio at the deepest point of the crack defect for pipelines with interacting corrosion and crack defects. Crack size, corrosion size and the axial distance between these two defects are the factors that have an impact on the growth of the fatigue crack, and so they are considered as the input of XGBoost models. Based on the synthetic samples from finite element modeling, it has been proved that the proposed approaches can effectively predict the SIF interaction impact ratio with relatively high accuracy. The crack propagation models are built based on the proposed XGBoost models, Paris’ law and corrosion growth model. Sensitivity analyses regarding corrosion initial depth and axial distance between defects are performed. The proposed method can support pipeline integrity management by linking the crack propagation model with corrosion size, crack size and the axial distance. The problem of how the interaction between corrosion and crack defects impacts crack defect growth is investigated.

## 1. Introduction

Pipelines are widely used to transport oil and gas products over long distances. Ensuring pipeline safety is a prerequisite for the transportation of fuels such as oil and natural gas. Researchers are committed to constructing more accurate and effective health management models and improving the integrity management system of pipelines. Researchers [1,2,3] summarized the existing models in the field of pipeline integrity management and pointed out that, although the current models consider the accuracy of inline inspection tools, they are still too ideal and challenging to accurately reflect the proper working conditions of the pipeline. Metal-loss corrosion defects are significant threats to pipeline integrity. Some researchers use stochastic processes to describe uncertainties associated with the degradation of wall thickness incurred by corrosion defects. Wang et al. [4] proposed a stochastic corrosion growth model using the geometric Brownian bridge process. Ossai et al. [5] used a non-homogeneous linear growth pure birth Markov model to predict the degradation of internal corrosion defects in oil and gas pipelines. Bazan and Beck [6] employed a Poisson square wave process to describe the corrosion growth rate and compared the proposed non-linear stochastic model with the linear corrosion growth model. Qin et al. [7] proposed a corrosion growth model based on Inverse Gaussian process and Markov Chain Monte Carlo simulation method. Pan et al. [8] also used Inverse Gaussian process to characterize the degradation process of defects. Peng et al. [9] proposed a Bayesian framework of Inverse Gaussian process models. Remaining useful life of pipelines with multiple defects was predicted in refs. [10,11,12]. Although these corrosion growth models take multiple corrosion defects into account, they hardly consider the interacting effects among these defects, let alone the interactions between different types of defects.

There are a number of papers investigating pipelines with interacting corrosion defects. Benjamin et al. [13,14] presented a detailed literature review of pipelines with interacting corrosion defects and a database of corroded pipe tests. Amandi et al. [15] proposed a finite element model combined with a curve fitting method to estimate the remaining strength of pipelines with interacting corrosion defects. Sun and Cheng [16] also implemented a 3D finite element model to investigate mechano-electrochemical interaction of multiple longitudinal corrosion defects. Soares et al. [17] presented a model to analyze the integrity of pipelines with interacting corrosion defects under internal pressure and thermal stresses. Chen et al. [18] used a nonlinear finite element model to study the failure pressure of X80 pipelines with interacting corrosion defects. Kuppusamy et al. [19] investigated the effect of interaction of corrosion defects on the buckling strength of pipelines. Assessing and managing crack defects is also a vital part of pipeline integrity management. The remaining useful life prediction for pipelines with a single crack defect was conducted in refs. [20,21,22]. As for pipelines with interacting crack defects, Zhang et al. [23] presented a numerical model and fatigue simulations to analyze the fatigue behaviors. The corrosive environment will affect the growth of the crack, which is called Stress Corrosion Cracking (SCC). Hu et al. [24] applied the Monte Carlo method to predict and evaluate SCC. Lu et al. [25] established an SCC crack growth model in a high pH environment and verified it through experiments. Sekhar [26] summarized the effects of various crack interactions. This study shows that it is necessary to include the analysis of the interaction coupling between crack defects. These studies are all about the interaction between different defects of the same type. However, the exploration of interaction impact between different types of defects is still lacking in the existing literature.

In pipelines, common pipeline defects, such as crack and corrosion, exist at the same time. Specifically, there is an interacting effect between the fatigue crack and corrosion defect in the same pipeline segment. Pipeline corrosion will change the strength of the pipeline in the surrounding area. If the corrosion and crack defects are adjacent, a certain interaction coupling will occur and impact the Stress Intensity Factor (SIF) of the crack surface, thereby affecting the propagation of fatigue crack. Therefore, the crack propagation model that considers the interaction between these two types of defects is conducive to formulating more accurate detection and maintenance strategies. Motivated by this need, this paper plans to study the interacting effects of corrosion and crack defects on pipeline crack propagation.

In this paper, a method was developed for predicting the propagation of fatigue crack for pipelines with interacting corrosion and crack defects. Crack length, crack depth, corrosion length, corrosion depth and the axial distance between the crack and corrosion defects are all considered when developing this method. The finite element models are built to obtain the SIF values with and without considering the interaction impact between these defects. The powerful regression model, XGBoost [27], is applied in this paper to predict the SIF interaction impact ratio. With synthetic data from finite element analysis modeling, two approaches are provided to fit and predict the SIF interaction impact ratio at the deepest point of the crack defect, considering the interaction between corrosion and crack defects. The first one uses the data samples to directly fit and predict the SIF interaction impact ratio with a XGBoost model. As for the second one, it is an indirect prediction approach. It fits the SIF with and without considering the interaction impact, respectively. Therefore, two XGBoost models are acquired in this approach. The prediction results from these two models are utilized to calculate SIF interaction impact ratio. SIF interaction impact ratio is defined as the ratio of the SIF considering the interaction impact divided by the SIF without considering the interaction impact. With the proposed XGBoost models and traditional crack and corrosion growth models, a crack propagation model is proposed for pipelines with interacting crack and corrosion defects, and simulation results are obtained for sensitivity analysis.

The novelty of this paper is three-fold: (1) it studies the interaction impact between different types of defects in pipelines, viz. crack and corrosion defects, depending upon the crack size, corrosion size and the axial distance between them; (2) it introduces SIF interaction impact ratio to describe the degree of the interaction impact and employs an advanced machine learning algorithm XGBoost to fit and predict the SIF interaction impact ratio; and (3) it proposes a method to predict the propagation of fatigue crack considering the interaction impact.

The rest of the paper is organized as follows. Section 2 presents the finite element analysis model for a pipeline with interacting corrosion and crack defects. Section 3 presents the proposed crack propagation model based on XGBoost. In Section 4, experimental results are obtained to analyze the interaction impact. Conclusions are presented in Section 5.

## 2. The Pipeline Finite Element Analysis Model

### 2.1. The FEA Model

**Figure 1:**
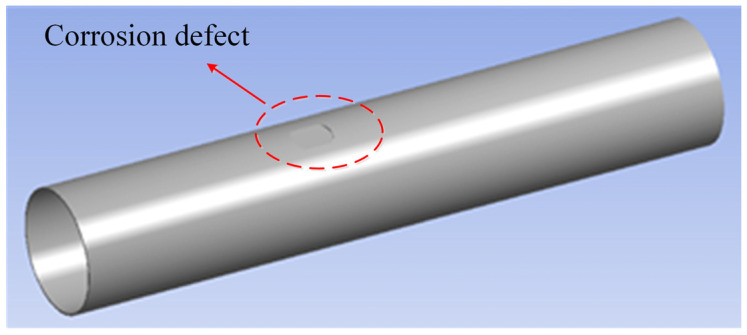
Geometric modeling of corroded pipeline.

**Figure 2:**
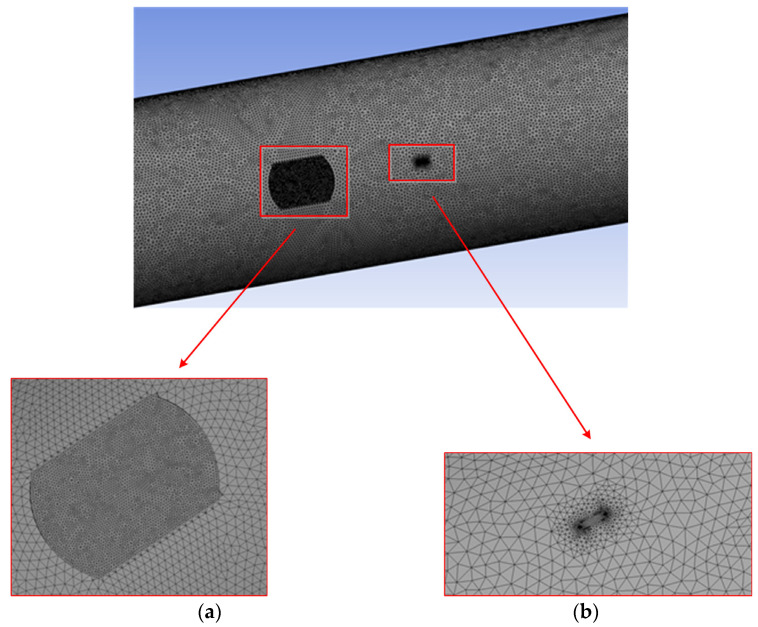
The grid division of pipeline. (**a**) Partial refinement of corrosion defect grid. (**b**) Partial refinement of grid at crack.

In this section, the finite element software ANSYS*^®^* is used to model the pipeline with interacting fatigue crack and external corrosion defects. In the modeling process, the pipeline models with and without corrosion defects are established, respectively, to analyze the interaction impact of corrosion defect on crack propagation. The material of the modelled pipeline is API 5L X70. The outside diameter of the pipeline is set as 914.4 mm, and the wall thickness is 15.875 mm. The internal pressure is assumed to be 1 MPa for modeling. The fatigue crack is modeled as a semi-elliptical shape with a length of 15.2 mm and a crack depth in the range of 2 mm–12 mm. The SIF values corresponding to the deepest point and edge point can be obtained through stress analysis. The internal pressure of the pipeline is 1 MPa. At the same time, there are cuboid corrosion defects on the outer surface of the pipeline, and the axial distance from the crack center to the corrosion center moves from 150 mm to 500 mm. The depth of the corrosion defect is from 2 mm to 14 mm, with an increment of 1 mm each time. The geometric modeling of a corroded pipeline is shown in Figure 1, and the finite element model built in this paper is shown in Figure 2.

### 2.2. Validation of FEA

Generally, fracture in engineering structures can be classified into three types: opening mode (I), sliding mode (II) and tearing mode (III), and SIF is used to reflect these modes. Compared with mode II and III, SIF corresponding to mode I is much larger, so the mode I SIF dominates the propagation of fatigue crack. In this paper, mode I SIF was only considered in the pipeline remaining useful life prediction. The method based on API579 for the partial verification of FEA model was employed. According to API579 criterion, the SIF of mode I of the pipeline is calculated as follows:

(1) $$K=\frac{pR_{i}^{2}}{R_{o}^{2}-R_{i}^{2}}\left[2G_{0}+2G_{1}\left(\frac{a}{R_{o}}\right)+3G_{2}{\left(\frac{a}{R_{o}}\right)}^{2}+4G_{3}{\left(\frac{a}{R_{o}}\right)}^{3}+5G_{4}{\left(\frac{a}{R_{o}}\right)}^{4}\right]\sqrt{\frac{\pi a}{Q}}$$

(2) $$Q=1.0+1.464{\left(\frac{a}{c}\right)}^{1.65},\;for\;\frac{a}{c}\leq 1$$

(3) $$G_{0}=A_{0,0}+A_{1,0}\beta +A_{2,0}\beta^{2}+A_{3,0}\beta^{3}+A_{4,0}\beta^{4}+A_{5,0}\beta^{5}+A_{6,0}\beta^{6}$$

(4) $$G_{1}=A_{0,1}+A_{1,1}\beta +A_{2,1}\beta^{2}+A_{3,1}\beta^{3}+A_{4,1}\beta^{4}+A_{5,1}\beta^{5}+A_{6,1}\beta^{6}$$

(5) $$\beta =\frac{2\phi }{\pi }$$

(6) $$G_{2}=\frac{\sqrt{2Q}}{\pi }\left(\frac{16}{15}+\frac{1}{3}M_{1}+\frac{16}{105}M_{2}+\frac{1}{12}M_{3}\right)$$

(7) $$G_{3}=\frac{\sqrt{2Q}}{\pi }\left(\frac{32}{35}+\frac{1}{4}M_{1}+\frac{32}{315}M_{2}+\frac{1}{20}M_{3}\right)$$

(8) $$G_{4}=\frac{\sqrt{2Q}}{\pi }\left(\frac{256}{315}+\frac{1}{5}M_{1}+\frac{256}{3465}M_{2}+\frac{1}{30}M_{3}\right)$$

(9) $$M_{1}=\frac{2\pi }{\sqrt{2Q}}\left(3G_{1}-G_{0}\right)-\frac{24}{5}$$

(10) $$M_{2}=3$$

(11) $$M_{3}=\frac{6\pi }{\sqrt{2Q}}\left(G_{0}-G_{1}\right)+\frac{8}{5}$$

where *p* is the internal pressure; *R_i_* is the internal radius; *R_o_* is the outer radius; *a* is the crack depth; *Q* is a parameter based on crack geometry; *G*_0_, *G*_1_, *G*_2_, *G*_3_, *G*_4_, *M*_1_, *M*_2_, *M*_3_, *A_i,j_* (i ∈ {0,1,2,3,4,5,6}, {j ∈ 0,1}), *β* are influence coefficients; *ϕ* is the included angle; *c* is the half crack length; and *K* is the mode I SIF.

**Figure 3:**
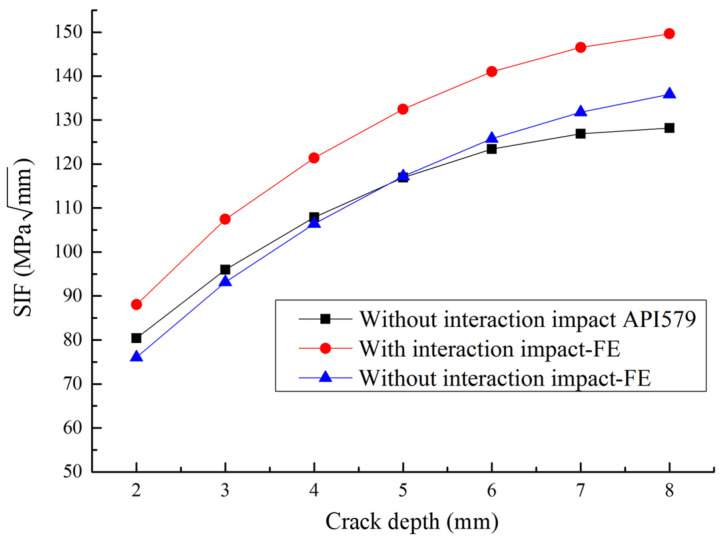
Comparison of pipeline SIF results.

The finite element simulation results are compared with the SIF results calculated according to API 579 criterion. The results are shown in Figure 3. It can be found from the figure that for the pipeline without corrosion defects, the SIF obtained by finite element simulation is very close to the results of theoretical calculation for a large portion of crack depth range, and the maximum error is less than 5%. The accuracy of finite element simulation is proved. Then, the SIF of pipeline with corrosion defects is studied. As is obtained from Figure 3, for the same crack depth, the SIF of the pipeline with corrosion defects is greater than that without corrosion defects. With the increase in crack depth, SIF also increases gradually. The comparison results demonstrate that there is an interacting impact of corrosion and crack defects on SIF values. Therefore, it is necessary to study the interacting impact between corrosion and crack defects.

## 3. The Proposed Crack Propagation Method Based on Extreme Gradient-Boosting Algorithm

### 3.1. The Extreme Gradient-Boosting Model

**Figure 4:**
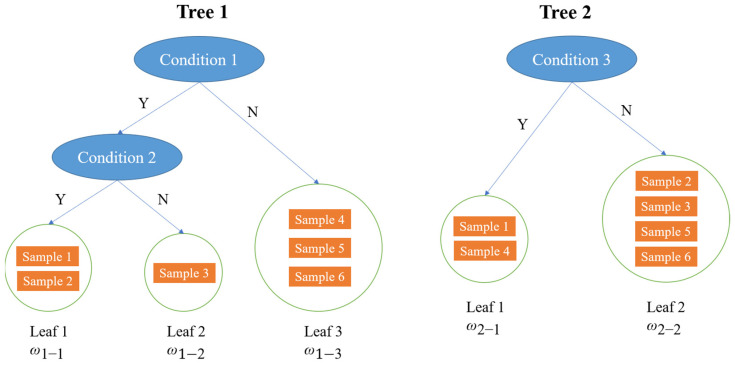
An example of CARTs.

Extreme Gradient Boosting (XGBoost) is an ensemble machine learning algorithm based on Decision Tree and uses Gradient Boosting as the framework. It is developed from Gradient-Boosting Decision Tree (GBDT). GBDT is an additive model based on boosting, which is a general ensemble method. It employs a forward stagewise algorithm for greedy learning in the training process. In each iteration, GBDT learns a Classification and Regression Tree (CART), where Figure 4 is an example of CARTs, to fit the residual error between the prediction result from previous CARTs and the actual value of the training dataset. In other words, it is there to build a model from the training dataset and create a second model to correct the residual error from the first model. Then, the models are added until the training dataset is predicted relatively accurately, or a maximum number of models is added.

Several optimization strategies are added into XGBoost model. Firstly, in order to improve computational accuracy, XGBoost uses the second-order derivative to optimize the objective function. Conversely, GBDT only uses the first-order derivative for optimization. In addition, the objective function of XGBoost utilizes regularization term to simplify the model and avoid overfitting. On the contrary, the GBDT does not have any regularization term in the objective function. XGBoost is able to automatically process default values and compute in parallel through a block storage structure, which cannot be implemented in GBDT. Since XGBoost has a high precision on the second-order derivative and fast parallel computation speed, it is very efficient in data processing and data modeling. In addition, XGBoost is relatively flexible, as it supports classification and regression, and it is able to provide customized objective function. XGBoost can be used with multiple programing languages and platforms. Therefore, XGBoost is widely used in the areas of data mining, recommender system and so on.

The objective function of XGBoost in the training process consists of two parts: loss function and regularization term:

(12) Obj(Θ)=L(Θ)+Ω(Θ)

where Θ is the parameters obtained from the training processing; L(Θ) is the training error, which denotes the matching degree of the model to the training dataset; Ω(Θ) is the regularization term, which represents the complexity of the model. Assuming that the training dataset is $S=\text{\{}(x_{1},y_{1}),\;(x_{2},y_{2}),\text{…},{(x}_{n},y_{n})\text{\}}$, the training error *L* can be expressed as the following equation:

(13) $$L=\overset{n}{\underset{i=1}{\sum }}l\left(y_{i},{\hat{y}}_{i}\right)$$

where $y_{i}$ and ${ŷ}_{i}$ are the target output and the predicted output of the *i*-th sample $x_{i}$ ($x_{i}\in R^{z},\;z$ is the number of features of the dataset), respectively, and *n* is the number of samples in the training dataset. For the proposed gradient-boosted machine, $l(y_{i},{ŷ}_{i})={\left(y_{i}-{ŷ}_{i}\right)}^{2}$. The objective is to minimize Obj(Θ), which means L(Θ) and Ω(Θ) should be relatively small. During the training process, it is required to balance the tradeoffs between bias and variance. Bias is controlled by L(Θ) and variance is controlled by Ω(Θ). L(Θ) and Ω(Θ) would be relatively large if underfitting. If overfitting, Ω(Θ) would also be relatively large, since the model is weak on scalability and stability. Assuming there are V CARTs in the model, then

(14) $${ŷ}_{i}=\overset{V}{\underset{v=1}{\sum }}f_{v}\left(x_{i}\right),\;f_{v}\in F$$

where F is the function space of all the CARTs in the model. $f_{v}{(x}_{i})$ represents the weight of the *i*-th sample falling on the leaf in the *v*-th tree. For the example in Figure 4, $f_{1}{(\mathrm{sample}2)=w}_{1\text{-}1}$, $f_{2}{(\mathrm{sample}2)=w}_{2\text{-}2}$, ${f\;(\mathrm{sample}2)=w}_{1\text{-}1}+w_{2\text{-}2}.$ Then, the model parameters that will be optimized from the training process are ${\Theta =\text{\{}f}_{1}{,\;f}_{2},\;\ldots {,\;f}_{V}\text{\}}$, where $f_{v}$ denotes the weight distribution of the samples falling on the leaf in the *v*-th tree. The objective function is shown in Equation (15):

(15) $$Obj=\overset{n}{\underset{i=1}{\sum }}l\left(y_{i},{ŷ}_{i}\right)+\overset{V}{\underset{v=1}{\sum }}\Omega \left(f_{v}\right)$$

Next, the objective function will be optimized in three steps. The first step is to use the second-order Taylor series expansion to optimize the loss function. The predicted values can also be expressed as

(16) $${{ŷ}_{i}}^{\left(u\right)}={{ŷ}_{i}}^{\left(u-1\right)}+f_{u}(x_{i})$$

which is the same as the expression of the GBDT. ${{ŷ}_{i}}^{\left(u\right)}$ is the predicted value of $x_{i}$ in tree *u* after the *i*-th iteration. Then, the objective function after the *i*-th iteration can be represented using Equation (17):

(17) $$Obj^{\left(u\right)}=\sum_{i=1}^{n}l(y_{i},{{ŷ}_{i}}^{\left(u-1\right)}+f_{u}(x_{i}))+\sum_{v=1}^{u}\Omega (f_{v})$$

Using the second-order Taylor series expansion, the loss function becomes

(18) $$\sum_{i=1}^{n}l(y_{i},{{ŷ}_{i}}^{\left(u-1\right)}+f_{u}(x_{i}))\approx \sum_{i=1}^{n}\left[l(y_{i},{{ŷ}_{i}}^{\left(u-1\right)})+g_{i}f_{u}(x_{i})+\frac{1}{2}h_{i}{f_{u}}^{2}(x_{i})\right]$$

where

(19) $$g_{i}=\frac{d(l(y_{i},{{ŷ}_{i}}^{\left(u-1\right)}))}{d({ŷ}^{\left(u-1\right)})}$$

(20) $$h_{i}=\frac{\partial^{2}(l(y_{i},{{ŷ}_{i}}^{\left(u-1\right)}))}{\partial {\left({ŷ}^{\left(u-1\right)}\right)}^{2}}$$

and the objective function is expressed in Equation (21):

(21) $$Obj^{\left(u\right)}\approx \sum_{i=1}^{n}\left[l(y_{i},{{ŷ}_{i}}^{\left(u-1\right)})+g_{i}f_{u}(x_{i})+\frac{1}{2}h_{i}{f_{u}}^{2}(x_{i})\right]+\sum_{v=1}^{u}\Omega (f_{v})$$

The second step is to optimize the regularization term by expanding the regularization term and removing the constant term. Since forward calculation is adopted in XGBoost, then the structure of the (*u* − 1)-th tree has been confirmed:

(22) $$l(y_{i},{{ŷ}_{i}}^{\left(u-1\right)})=constant$$

(23) $$\begin{array}{c} \sum_{v=1}^{u}\Omega (f_{v})=\Omega (f_{u})+\sum_{v=1}^{u-1}\Omega (f_{v})\\ \;=\Omega (f_{u})+constant \end{array}$$

Then the objective function is expressed as follows:

(24) $$\begin{array}{c} Obj^{\left(u\right)}\approx \sum_{i=1}^{n}\left[l(y_{i},{{ŷ}_{i}}^{\left(u-1\right)})+g_{i}f_{u}(x_{i})+\frac{1}{2}h_{i}{f_{u}}^{2}(x_{i})\right]+\Omega (f_{u})+constant\\ \;=\sum_{i=1}^{n}\left[g_{i}f_{u}(x_{i})+\frac{1}{2}h_{i}{f_{u}}^{2}(x_{i})\right]+\Omega (f_{u})+[\sum_{i=1}^{n}l(y_{i},{{ŷ}_{i}}^{\left(u-1\right)})+constant]\\ \;=\sum_{i=1}^{n}\left[g_{i}f_{u}(x_{i})+\frac{1}{2}h_{i}{f_{u}}^{2}(x_{i})\right]+\Omega (f_{u})+constant \end{array}$$

After removing the constant term, the simplified objective function is

(25) $$Obj^{\left(u\right)}\approx \sum_{i=1}^{n}\left[g_{i}f_{u}(x_{i})+\frac{1}{2}h_{i}{f_{u}}^{2}(x_{i})\right]+\Omega (f_{u})$$

The last step of the optimization process is to merge the coefficients of the first-degree term and the quadratic term. Regarding the definition of a tree, the weight vector of leaves is set as $w\;\in \;R^{T}$and the mapping relationship between the leaves (viz. the structure of the tree) is defined as $q:R^{Z}\to \{1,\;2,\;3,\;\ldots ,\;T\}$ where *T* is the number of leaves in the tree. Then, q(x) denotes the location of the leaf, for sample *x*. For the example in Figure 4, q(sample2)=1 in tree 1, q(sample2)=2 in tree 2. $f_{t}(x)$ can be represented by

(26) $$f_{u}(x)=w_{q(x)}$$

Here, the number of leaves *T* and smoothness of leaf weight (viz. L2 norm of leaf weights) are used to describe the complexity of the tree, so

(27) $$\Omega (f_{u})=\gamma T+\frac{1}{2}\lambda \sum_{j=1}^{T}{w_{j}}^{2}$$

For the example in Figure 4, $\Omega (f_{1})=\gamma 3+\frac{1}{2}\lambda \left({w_{1-1}}^{2}+{w_{1-2}}^{2}+{w_{1-3}}^{2}\right)$, $\Omega (f_{2})=\gamma 2+\frac{1}{2}\lambda \left({w_{2-1}}^{2}+{w_{2-2}}^{2}\right)$. $I_{j}=\{i\;|\;q(x_{i})=j\}$ is the instance set in leaf *j*, *j* = 1, 2, …, *T*. Grouping all the training samples based on leaves and utilizing Equations (26) and (27), then, the objective function is

(28) $$Obj^{\left(u\right)}\approx \sum_{j=1}^{T}\left[(\sum_{i\in I_{j}}g_{i})w_{j}+\frac{1}{2}(\sum_{i\in I_{j}}h_{i}+\lambda )w_{j}^{2}\right]+\gamma T$$

At last, merging the first-degree term and the quadratic term, then

(29) $$Obj^{\left(u\right)}\approx \sum_{j=1}^{T}\left[G_{j}w_{j}+\frac{1}{2}(H_{j}+\lambda )w_{j}^{2}\right]+\gamma T$$

where

(30) $$G_{j}=\sum_{i\in I_{j}}g_{i},H_{j}=\sum_{i\in I_{j}}h_{i}$$

For each leaf *j*, the objective function is expressed as follows:

(31) $$f(w_{j})=G_{j}w_{j}+\frac{1}{2}(H_{j}+\lambda )w_{j}^{2}$$

As the objective function of each leaf in the overall objective function is independent, then the overall objective function will achieve the minimum value when each leaf’s objective function is minimized. The optimal solution of the quadratic function of one variable is

(32) $$w_{j}^{*}=-\frac{G_{j}}{H_{j}+\lambda }$$

At this point, each leaf weight is optimized, and the overall objective function achieves its optimal value, viz. the minimum value:

(33) $$Obj^{*}=-\frac{1}{2}\sum_{j=1}^{T}\frac{G_{j}}{H_{j}+\lambda }+\gamma T$$

**Figure 5:**
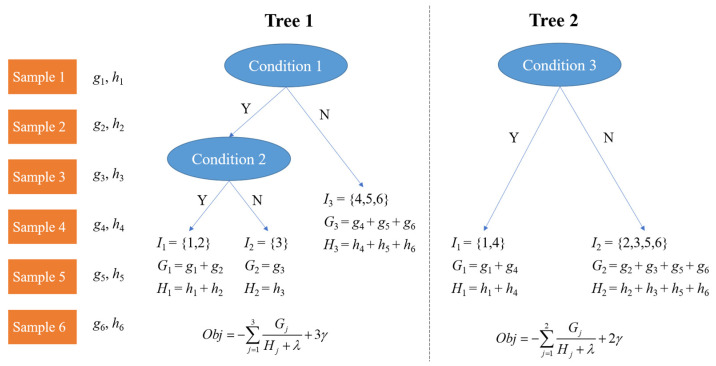
Objective function of the example in Figure 4.

The structure of the tree is also the best at this time. The optimal objective functions of Figure 4 are shown in Figure 5. The fewer objective functions there are, the better the tree structures are.

In the actual training process, finding the optimal split point is a key problem. The applicable methods include greedy algorithm, approximate algorithm, weighted quantile sketch and sparsity-aware split finding. The greedy algorithm is the most commonly used.

### 3.2. The Proposed Model Based on XGBoost

In the proposed method, the scikit-learn wrapper interface for XGBoost was utilized to construct models to predict the SIF interaction impact ratio at the deepest point of the crack defect for pipelines with corrosion and crack defects. Based on the observations from finite element modeling, the size of crack and corrosion defects, and the axial distance between them, can affect SIF results. Therefore, the input variables of the proposed model are the length and depth of the crack defect, the length and depth of the corrosion defect, and the axial distance between the crack and the corrosion defects. The output variable is the interaction impact ratio *α*. The input and output variables are shown in Table 1. The crack length is assumed in the range of 15.2 mm–76.0 mm, and the crack depth is in the range of 2 mm–12 mm. The axial distance between the corrosion and crack defects is from 150 mm to 500 mm. The depth of the corrosion defect is from 2 mm to 14 mm.

In this paper, two approaches are provided to fit *α*. The first one is to directly construct a XGBoost model to predict *α*. The second one is to construct two XGBoost models to fit SIF values with and without considering the interaction impact, which are K* and *K*, respectively. Then, the interaction impact ratio can be calculated with the formula *α* = K*/*K*. It is worth noting that only crack length and crack depth have an impact on SIF without considering interaction impact. Then, in the process of fitting *K*, there are only two input variables, viz. crack depth and crack length.

The samples used for modeling are synthetic data from finite element modeling. In total, 385 pieces of data are generated. A share of 80% of these data was randomly selected as the training set. The remaining data are the testing set. The scikit-learn API for XGBoost regression has a lot of parameters to set. In this paper, five parameters are selected for parameter tuning to get the best model structure and parameters: the number of gradient-boosted trees, the maximum depth of a tree, the minimum sum of instance weight needed in a child, *L*1 and *L*2 regularization terms on weights. The adjusting ranges for these five parameters are shown in Table 2. Increasing the maximum depth of a tree will make the model more complex, and it will be more likely to overfit, so the maximum value for this parameter is set to 10 in this paper. If the sum of instance weight in a leaf node is less than the minimum sum of instance weight needed in a child, the building process will stop further partitioning. Regarding the *L*1 and *L*2 regularization terms on weights, increasing their values will make the model more conservative. The learning rate is set at 0.1, which updates the weights to prevent overfitting and makes the boosting process more conservative. For the other parameters, such as the initial prediction score of all instances (global bias), minimum loss function required to make a further partition on a leaf node of the tree, etc., the default values in the scikit-learn API are applied.

**Figure 6:**
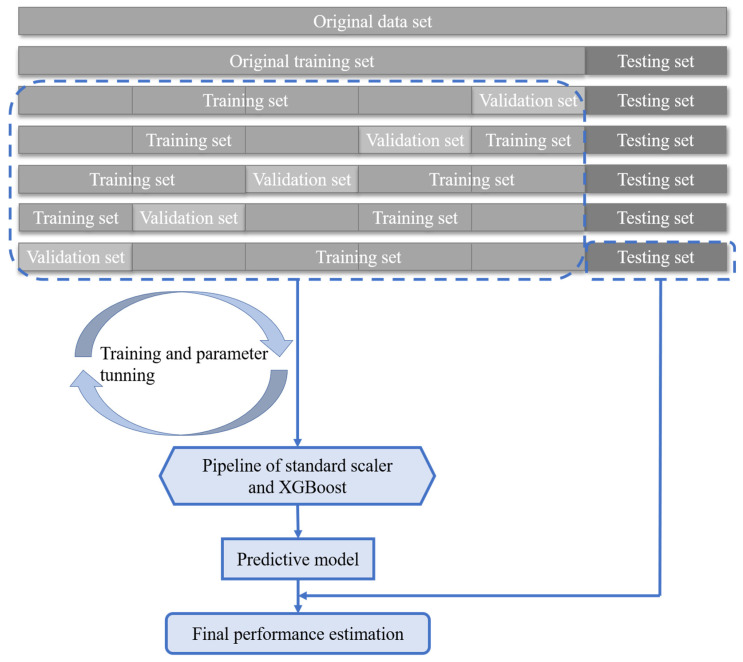
The procedure of the proposed algorithm.

In the training process, a grid search method with 5-fold cross-validation was applied to select the best combination of the tuning parameters based on the determination coefficient $R^{2}$, which describes the goodness of fit of the current trained model. In other words, the original training set was re-segmented into the training set and validation set with the ratio of 4:1 five times, as shown in Figure 6. For each combination of the tuning parameters, the training set was used to train the model, and the validation set was used to evaluate the model’s performance five times and compute the average performance, viz. average $R^{2}$, with these five times’ results. This method can reduce training bias and improve the model’s stability. After all the combinations’ results are obtained, the model with the highest $R^{2}$ has the best combination of the tuning parameters. The values of $R^{2}$ are between 0 and 1. A value much closer to 1 indicates the regression model has a higher fitting degree.

In the actual training process, a pipeline of transforms with a final estimator (viz. model to be fitted) is utilized. This method is to sequentially apply a list of transforms and a final model. Intermediate steps of the pipeline must implement fit and transform methods, while the final model only needs to implement the fit method. In this paper, a pipeline consisting of a standard scaler and an XGBoost model is applied. The standard scaler is to normalize data to make its features have zero mean and unit variance. The standard scaler fits to the training set and transforms the training set and validation set.

The overall process of the first approach for constructing the XGBoost model is as follows:

Step 1. Randomly split the samples (output variable is SIF interaction impact ratio *α*) into training set and testing set with the ratio 8:2.

Step 2. Employ a pipeline consisting of a standard scaler and an XBoost model to the original training set for training. In detail, the 5-fold cross-validated grid search method is applied to the original training set to select the best model structure and parameters among all the combinations of the tuning parameters. The model with the highest $R^{2}$ is the best model. The best model is then saved and can be directly applied to new data to acquire prediction values.

Step 3. Feed the testing set to the trained model to obtain the value of $R^{2}$, which indicates the ability fitting to new data with the trained model. The closer that $R^{2}$ is to 1, the better structured the model is. If the value is close to 1, then the trained model can be used to directly predict interaction impact ratio *α*.

Similarly, the overall process of the second approach for constructing the two XGBoost models is:

Step 1. Randomly split the samples (output variables are SIF values with and without considering interaction impact, viz. K* and *K*) into training set and testing set with the ratio 8:2.

Step 2. When the output variable is *K*, the input’s variables are crack depth and crack length, and employ a pipeline of a standard scaler and an XGBoost model to the original training set for training. In the same way, the 5-fold cross-validated grid search method is applied to the original training set to select the best model. The best model is saved to predict *K*.

Step 3. When the output variable is K*, the input includes all the five input variables and employs a pipeline of a standard scaler and an XGBoost model to the original training set for training. In the same way, the 5-fold cross-validated grid search method is applied to the original training set to select the best model. The best model is then saved to predict K*.

Step 4. Respectively, feed the two testing sets to the two trained models to obtain the values of $R^{2}$. If the two values of $R^{2}$are close to 1, then the two trained models can be used to predict SIF with and without considering interaction impact, respectively.

Step 5. Calculate predicted interaction impact ratio *α* on the testing set with predicted *K* and K* and compare the predicted values with the target ratio values by calculating $R^{2}$.

### 3.3. The Pipeline Corrosion and Fatigue Crack Growth Models

In the proposed model, corrosion defect is assumed to grow linearly. The growth of the corrosion depth is characterized by

(34) $$d\left(t\right)=d_{0}+g_{d}t$$

where *d*_0_ represents the corrosion initial depth, *g_d_* is the growth rate of corrosion depth, and *t* is the propagation time. The corrosion depth is used as the input variable in the XGBoost model to calculate the SIF interaction impact ratio. In this paper, the corrosion depth growth rate is assumed to be 0.3 mm/year [11].

Pipeline fatigue crack growth is predicted using the physics-based methods governed by Paris’ law, which was employed in [28,29,30]. Based on Paris’ law and the proposed model for evaluating the SIF interaction impact between corrosion and crack defects, the fatigue crack growth model is introduced in the following equation:

(35) $$da/dN=C{\left(\Delta Κ\alpha \right)}^{m}$$

where *da*/*dN* is crack growth rate; *a* is crack depth; *N* is the number of loading cycles; α is the SIF interaction impact ratio; and Δ*K* is the range of SIF. *C* and *m* are material-related model parameters, which can be estimated via experiments. In this paper, it is assumed that model parameters *C* = 5 × 10^−12^, *m* = 3 [21]. Methods based on FE and XGBoost models are employed to calculate the SIF and SIF interaction impact ratio at the deepest point of the fatigue crack. In this study, this paper focuses on the crack depth growth, since the length is mostly unchanged.

## 4. Results

**Figure 7:**
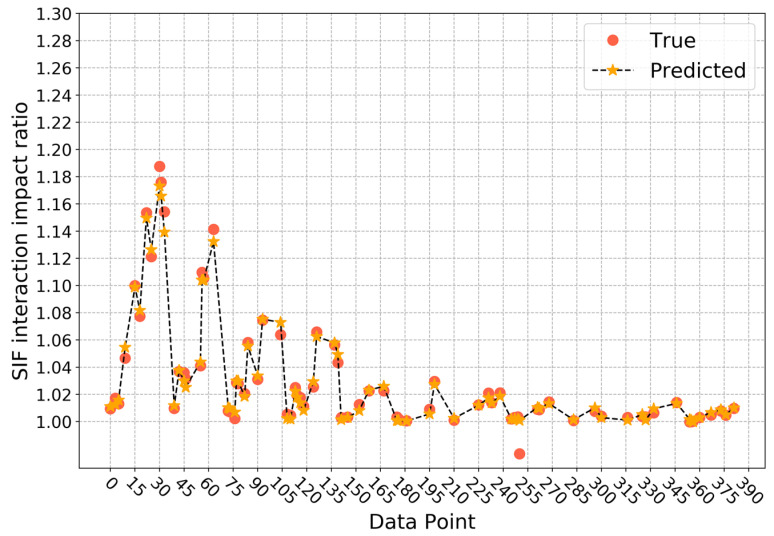
Prediction results of SIF interaction impact ratio based on approach 1.

When directly fitting the SIF interaction impact ratio, the average determination coefficient on the validation sets during cross validation is 0.9935, and the standard deviation is 0.0041. Thus, it can be seen that the trained model has a relatively high stability. The prediction result on the testing set is as Figure 7 shows. On the testing set, the determination coefficient $R^{2}$ is 0.9876, which means the developed model can accurately predict the SIF interaction impact ratio. At this point, the number of gradient-boosted trees is 110, the maximum depth of a tree is 6, the minimum sum of instance weight needed in a child is 1, and the *L*1 and *L*2 regularization terms on weights are 0.05 and 0.1, respectively.

**Figure 8:**
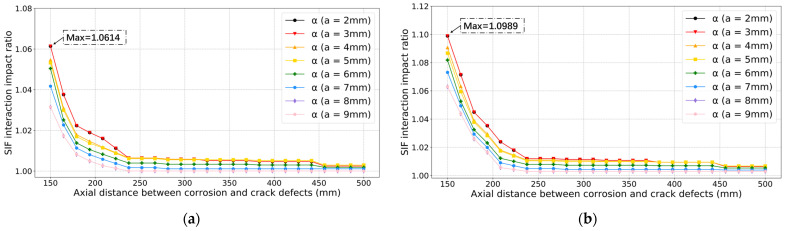

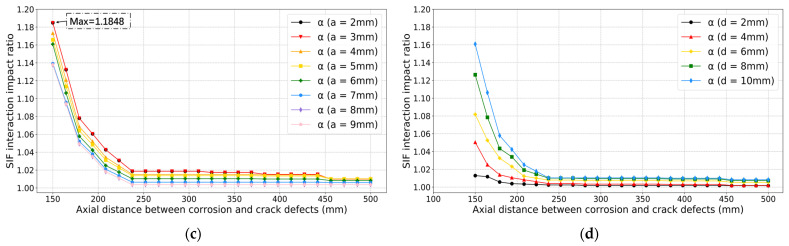
Prediction results of SIF interaction impact ratio based on approach 1. Corrosion depth *d*: (**a**) 4 mm; (**b**) 6 mm; (**c**) 10 mm; (**d**) Comparison results for different corrosion depths when crack depth *a* is 6 mm.

The prediction results of the SIF interaction impact ratio are shown in Figure 8. As observed in Figure 8, it can be found that the interaction impact ratio decreases as the crack depth *a* increases. From Figure 8a to Figure 8c, as corrosion depth increases from 4 mm to 10 mm, the SIF interaction impact ratio increases a lot when the axial distance between two corrosion and crack defects remains the same. Thus, the corrosion depth does affect the SIF interaction impact ratio a lot. The highest SIF interaction impact ratio in Figure 8c is 1.1848, which means it is necessary to consider the interaction impact between these two defects in the crack propagation process. The comparison results for different corrosion depths when crack depth is equal to 6 mm are shown in Figure 8d. From all these four figures, it can be found that the SIF interaction impact ratio is overall decreasing as the axial distance increases. These ratios first decrease quickly when the axial distance is smaller than around 175 mm and then decrease relatively slowly when the axial distance is in the range of 175 mm and 240 mm. When the axial distance is bigger than 240 mm, the decreasing speed is getting even smaller. This is because the corrosion defect moves away from the stress concentration zone of the crack defect.

**Figure 9:**
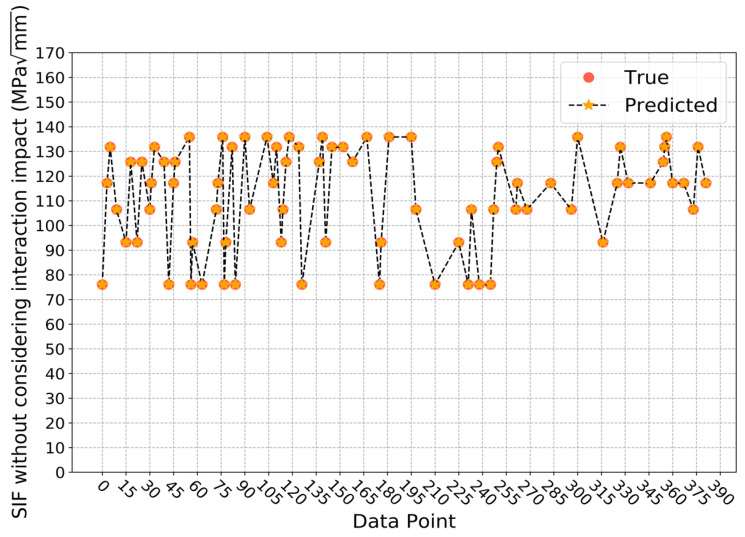
Prediction results of SIF values without considering interaction impact based on approach 2.

**Figure 10:**
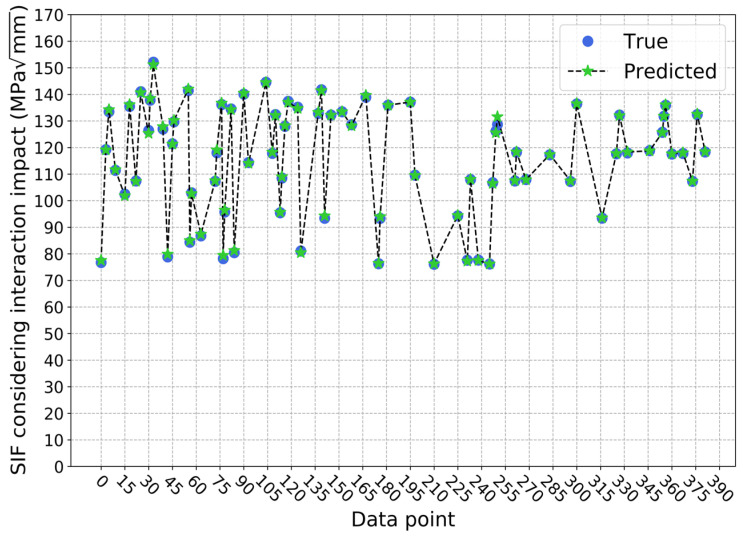
Prediction results of SIF values considering interaction impact based on approach 2.

**Figure 11:**
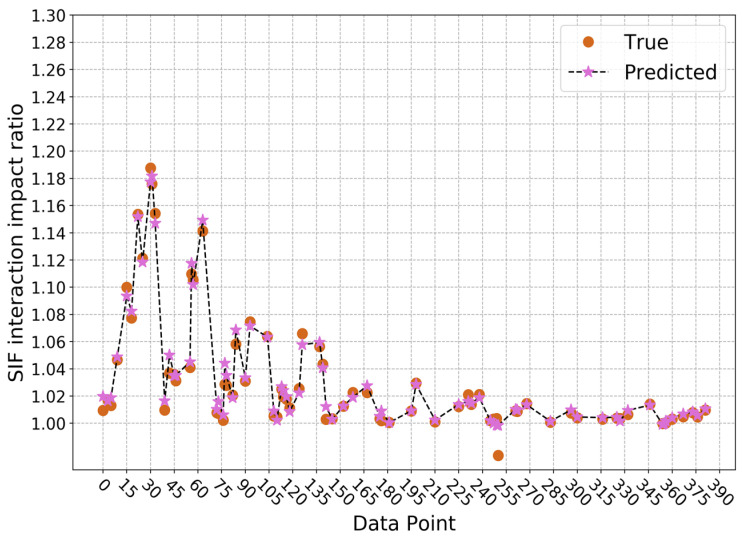
Prediction results of SIF interaction impact ratio based on approach 2.

For the second approach, the average determination coefficient on the validation sets is 1.0000 and the standard deviation is 0.0000 when predicting SIF values without considering interaction impact, which means the trained model is relatively stable. Here, the number of gradient-boosted trees is 110, the maximum depth of a tree is 3, the minimum sum of instance weight needed in a child is 1, and the *L*1 and *L*2 regularization terms on weights are both 0.05. The prediction result on the testing set is shown in Figure 9, where the determination coefficient $R^{2}\;$is 1.0000. When considering interaction impact, the average determination coefficient on the validation sets is still 0.9998, and the standard deviation is still 0.0001. However, the selected structure and parameters of the model are different. The number of gradient-boosted trees is 110, the maximum depth of a tree is 4, the minimum sum of instance needed in a child is 2, and the *L*1 and *L*2 regularization terms on weights are 0.1 and 1, respectively. On the testing set, the determination coefficient $R^{2}$ is 0.9992, and the prediction result is as displayed in Figure 10. It can be seen that for these two predictive models, the performance is quite stable on the validation sets and very accurate on the testing set. Therefore, it can be concluded that these two XGBoost models can predict SIF values with and without considering interaction impact efficiently and accurately. Furthermore, this indicates these two models are able to predict interaction impact ratio efficiently and accurately, since the ratio is calculated from the predicted results of these two models. After the predictive results are obtained from these two models, the SIF interaction impact ratio *α* can be calculated with the equation K*/*K*. For the testing set in this paper, the result is shown in Figure 11. At this time, the determination coefficient $R^{2}\;$of the SIF interaction impact ratio on the testing set is 0.9852. From the experimental result, it can also be concluded that the two trained XGBoost models can predict SIF interaction impact ratio at the deepest point of the crack defect, considering the interaction between corrosion and crack defects accurately and efficiently.

**Figure 12:**
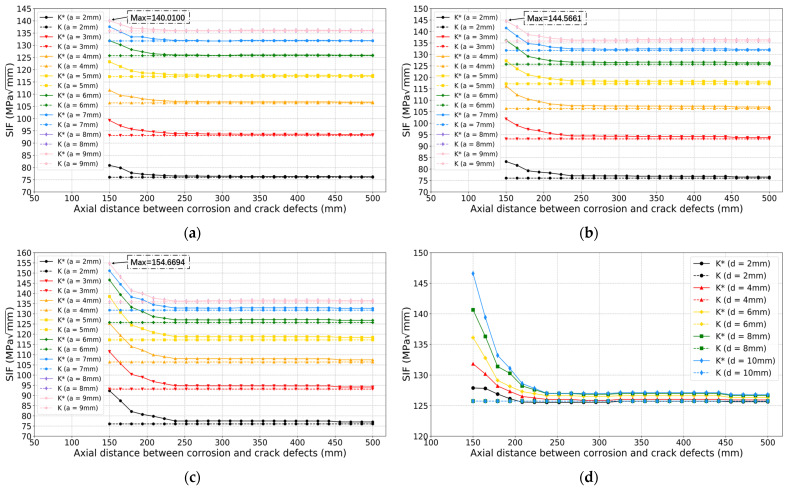
The comparison of SIF results based on approach 2. Corrosion depth *d*: (**a**) 4 mm; (**b**) 6 mm; (**c**) 10 mm; (**d**) Comparison results for different corrosion depths when crack depth *a* is 6 mm.

The comparison results of SIF values with and without considering interaction impact are shown in Figure 12. When increasing the crack depth *a* from 2 mm to 9 mm, K* and *K* values both gradually increase as expected. From Figure 12a to Figure 12c, K* values gradually increase, while *K* values remain the same as the corrosion depths increases. From the observations of these in Figure 12d, K* and *K* values have relatively big differences when the axial distance between two defects is smaller than around 240 mm.

**Figure 13:**
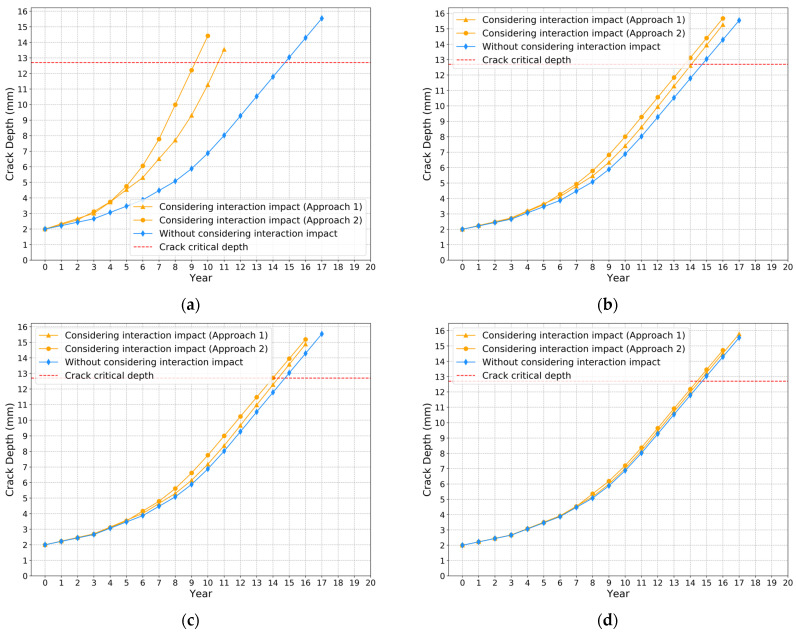
Investigations of the interaction impact on crack depth growth for different axial distances. Axial distance: (**a**) 150 mm; (**b**) 200 mm; (**c**) 300 mm; (**d**) 500 mm.

**Figure 14:**
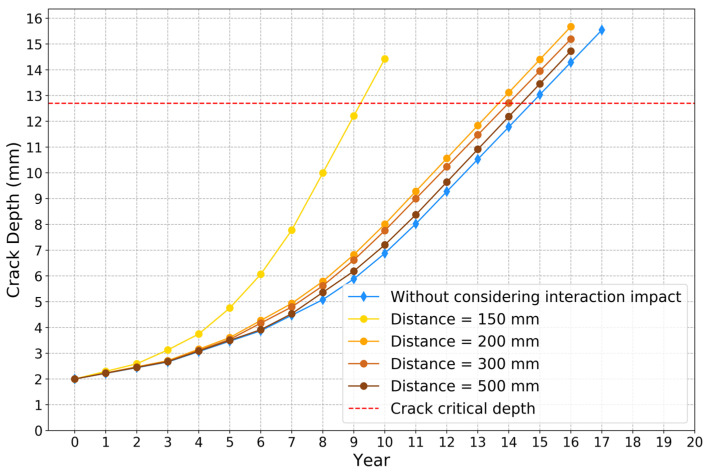
Crack depth growth curves for different distances based on approach 2.

The crack propagation models are built based on the proposed XGBoost model and Paris’ law. The crack initial depth is set at 2 mm, since SIF interaction impact ratio is relatively large when crack depth is small. The corrosion initial depth is assumed at 6 mm. Figure 13a–d show the comparison results of crack depth growth models for different axial distances between two defects using approach 1 and 2, respectively. The red dash lines represent the crack critical depth, which is approximately 80% of the wall thickness. When the crack depth exceeds the crack critical depth, it is considered a failure. The comparison results shown in Figure 13 indicate that the crack depth predicted by approach 2 reaches the threshold more quickly than approach 1. The comparison results of the crack depth propagation curves for different axial distances based on approach 2 are shown in Figure 14. If the interaction impact between two defects is not considered, it takes about 14.8 years to fail. Meanwhile, when considering the interaction impact between two defects by implementing the proposed model, the failure time changes from 9.2 to 13.8, 14.0 and 14.5 years, as the axial distance changes from 150 to 200, 300 and 350 mm. There is a big difference between the two crack propagation curves in Figure 13a, since the corrosion defect is in the stress concentration zone (axial distance smaller than 175 mm).

**Figure 15:**
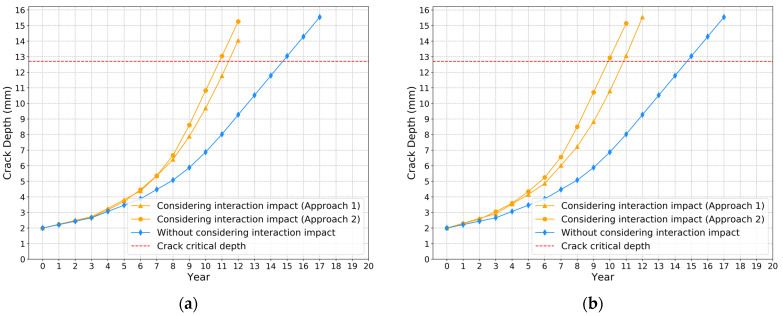

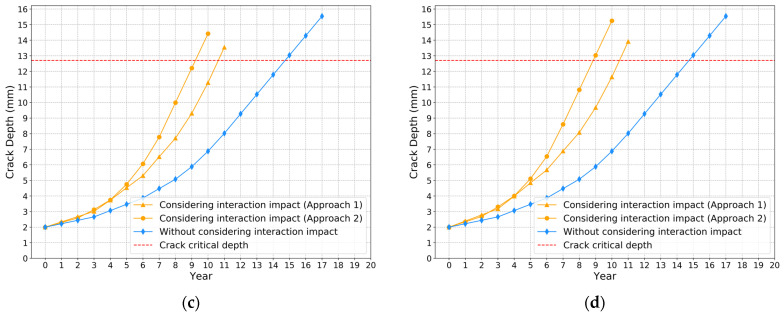
Investigations of the interaction impact on crack depth growth. Corrosion initial depth: (**a**) 2 mm; (**b**) 4 mm; (**c**) 6 mm; (**d**) 8 mm.

**Figure 16:**
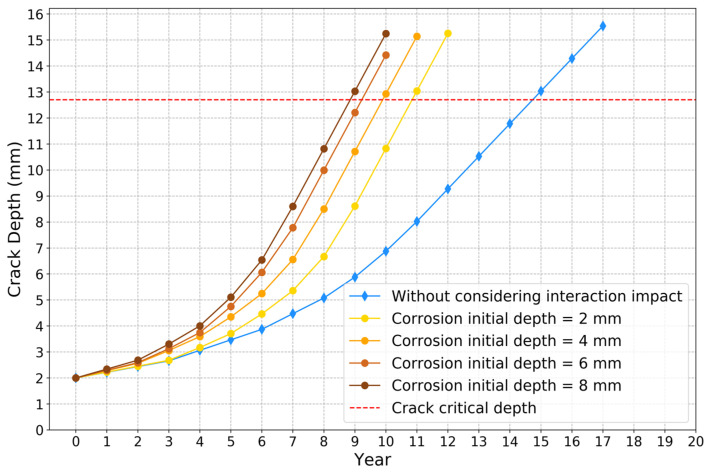
Crack depth growth curves for different corrosion initial depths based on approach 2.

To perform sensitivity analysis regarding corrosion initial depth, the aggressive case was studied, in which the axial distance was set at 150 mm. The corrosion initial depth varies from 2 mm to 8 mm. Figure 15a–d show the results of crack depth growth models for different corrosion initial depths. These figures indicate that the crack depth grows more quickly using approach 2 than approach 1. The comparison results of the crack depth propagation curves for different corrosion initial depths based on approach 2 are shown in Figure 16. If not considering the interaction impact between two defects, it also takes about 14.8 years to fail. From the comparison results in Figure 15 and Figure 16, the time to reach critical crack depth is 10.8, 9.9, 9.2 and 8.8 years, respectively, as the corrosion initial depth changes from 2 to 3, 6 and 8 mm. From the experimental results obtained from Figure 13, Figure 14, Figure 15 and Figure 16, it can be concluded that the interaction impact between corrosion and crack defects affects the propagation of fatigue crack a lot. Thus, it is necessary to consider the SIF interaction impact ratio in the remaining useful life prediction, especially when the corrosion defect is in the stress concentration zone.

## 5. Conclusions

The existing reported work only focuses on pipeline life prediction with single or multiple defects of the same type. The interaction impacts between different types of defects are not considered. In this work, the interaction impacts between crack and corrosion defects were studied, and a fatigue crack propagation method considering these impacts was proposed based on XGBoost models and Paris’ law. Crack size, corrosion size, and the axial distance between these two defects were all considered in the proposed method. In addition, this paper introduced SIF interaction impact ratio to describe how the corrosion defect affects the stress concentration zone of the fatigue crack. Two approaches were implemented for SIF interaction impact ratio prediction. The first one directly fitted and predicted SIF interaction impact ratio with the synthetic samples from finite element modeling. The second one fitted and predicted the SIF with and without considering interaction impacts, respectively, and then calculated the SIF interaction impact ratio. Examples were used to demonstrate the proposed method. The determination coefficients of these two approaches on the testing sets were 0.9876 and 0.9852, respectively, which was quite close to 1. Therefore, it can be concluded that the developed method can predict fatigue crack growth accurately. Several key findings are listed below:

The SIF interaction impact ratio decreases as the crack depth increases. It increases as the corrosion depth increases.

The SIF interaction impact ratio is gradually decreasing as the axial distance increases. This ratio is relatively large when the axial distance is smaller than 240 mm.

The time to reach critical crack depth decreases as the corrosion initial depth increases or the axial distance decreases.

The method developed in this paper can support the decision making in pipeline integrity planning, especially when the corrosion defect is relatively close to the crack defect. However, the proposed method only considered the interacting impact between two defects. More efficient crack and corrosion propagation models considering more than two defects are desired in future research. Another research topic is to develop crack propagation models for different types of crack shapes instead of semi-elliptical shapes.

## Figures and Tables

**Table 1:** Input and output variables of the proposed XGBoost models.

Input Variables	Output Variables
Crack length	SIF considering interaction impact (K*)
Crack depth	SIF without considering interaction impact (*K*)
Corrosion length	Interaction impact ratio (*α*)
Corrosion depth	
Axial distance between crack and corrosion defects	

**Table 2:** Parameter tuning for XGBoost models.

Parameters	Adjusting Ranges
Number of gradient-boosted trees	{40,50,60,70,80,90,100,110}
Maximum depth of a tree	{3,4,5,6,7,8,9,10}
Minimum sum of instance weight needed in a child	{1,2,3,4,5,6}
*L*1 regularization term on weights	{0.05,0.1,1,2,3}
*L*2 regularization term on weights	{0.05,0.1,1,2,3}

## Data Availability

The data presented in this study are available on request from the corresponding author.

## Nomenclature

*p*	pipeline internal pressure
*a*	crack depth
*c*	half crack length
*R_i_*	pipeline internal radius
*R_o_*	pipeline outer radius
*Q*	crack geometry parameter in API 579
*G_0_*, *G_1_*, *G_2_*, *G_3_*, *G_4_*, *M_1_*, *M_2_*, *M_3_*, *A_i,j_* (i ∈ {0,1,2,3,4,5,6}, j ∈ {0,1}), *β*	influence coefficients in API 579
*ϕ*	included angle in API 579
*Obj*	objective function of XGBoost
Θ	parameters obtained from the training processing in XGBoost
L	training error in XGBoost
Ω	regularization term in XGBoost
*S*	training dataset
*l*	training error of each sample in XGBoost
$x_{i}$	*i*-th sample
$y_{i}$	target output of the *i*-th sample
${ŷ}_{i}$	predicted output of the *i*-th sample
*z*	number of features in the dataset
*n*	number of samples in the training dataset
*V*	number of classification and regression trees in XGBoost
*v*	*v*-th classification and regression tree in XGBoost
$f_{v}$	weights of samples falling on the leaf in the *v*-th tree
*F*	function space of all the classification and regression trees in XGBoost
$g_{i}$	first-order derivative of training error for *i*-th sample in XGBoost
$h_{i}$	second-order derivative of training error for *i*-th sample in XGBoost
*w*	weight vector of leaves in classification and regression tree
*T*	number of leaves in classification and regression tree
*q*	mapping relationship between the leaves in classification and regression tree (viz. the structure of the tree)
*γ*	coefficient for number of leaves in regularization term in XGBoost
*λ*	coefficient for L2 norm of leaf weights in regularization term in XGBoost
$I_{j}$	instance set in leaf *j*
*G_j_*	sum of first-order derivatives of training error for leave *j* in XGBoost
*H_j_*	sum of second-order derivatives of training error for leave *j* in XGBoost
*K*	SIF without considering interaction impact
*K**	SIF considering interaction impact
*α*	interaction impact ratio
*d*	corrosion depth
*d_0_*	corrosion initial depth
*g_d_*	growth rate of corrosion depth
*t*	propagation time
*m, C*	material parameters in Paris’ law
*N*	loading cycles
Δ*K*	the range of SIF
